# Is Total Joint Arthroplasty an Effective and Safe Option for Psoriatic Arthritis Patients? A Scoping Review

**DOI:** 10.3390/jcm13185552

**Published:** 2024-09-19

**Authors:** Jacopo Ciaffi, Lorenzo Bianchi, Alberto Di Martino, Cesare Faldini, Francesco Ursini

**Affiliations:** 1 Medicine & Rheumatology Unit, IRCCS Istituto Ortopedico Rizzoli, 40136 Bologna, Italy; lorenzo.bianchi12@studio.unibo.it (L.B.); francesco.ursini2@unibo.it (F.U.); 2 Department of Biomedical and Neuromotor Sciences (DIBINEM), University of Bologna, 40126 Bologna, Italy; alberto.dimartino@ior.it (A.D.M.); cesare.faldini@ior.it (C.F.); 3 1st Orthopaedic and Traumatologic Clinic, IRCCS Istituto Ortopedico Rizzoli, 40136 Bologna, Italy

**Keywords:** psoriatic arthritis, outcomes, complications, arthroplasty, surgery, review

## Abstract

Recent advancements in the treatment of psoriatic arthritis (PsA) have improved patient outcomes, but many still experience disease progression, potentially leading to joint replacement surgery. In this scoping review, we examine the relationship between PsA and orthopedic surgery, focusing on the risks and temporal trends of total hip arthroplasty (THA) and total knee arthroplasty (TKA), the prevalence of postoperative complications, and the effectiveness of these procedures in PsA. The included studies suggest that PsA patients have an overall higher risk of undergoing THA and TKA compared to the general population, but with temporal trends showing a decreased risk for patients diagnosed in recent years. Acute complications, such as renal failure, stroke, and postoperative infections, may be more common in PsA patients than in those with osteoarthritis after THA and TKA. No significant differences were found in pain, function, or satisfaction between PsA, skin psoriasis, and osteoarthritis patients after THA. A key conclusion from our review is the need to strengthen the collaboration between rheumatologists and orthopedic surgeons, as interdisciplinary evaluation is crucial for improving the outcomes of PsA patients undergoing orthopedic surgery.

## 1. Introduction

In recent years, the advancements in pharmacological therapy and the treat-to-target approach have led to a significant shift in how psoriatic arthritis (PsA) is treated [1]. Biologic and targeted synthetic disease-modifying antirheumatic drugs (bDMARDs and tsDMARDs) have transformed the management of PsA patients, significantly improving functional abilities and quality of life [2]. Despite these treatment options, a substantial number of patients do not achieve minimal disease activity [3,4]. For these patients, structural damage may accumulate, leading to progressive functional impairment [5].

A critical issue that arises with disease progression in PsA is the increased need for joint replacement surgery, particularly total hip arthroplasty (THA) and total knee arthroplasty (TKA) [6,7]. This need is increased by the high prevalence of osteoarthritis (OA) in PsA patients, with a risk of developing OA that is 86% higher than in the general population and 68% higher than in psoriasis patients [8]. Importantly, PsA patients exhibit a higher risk of OA even prior to PsA diagnosis, suggesting that the increased need for joint replacement can result from both inflammatory and degenerative joint changes [8]. In the general population, the volume of THA and TKA has increased in the past decades, with projections indicating a rising demand in the coming years [9,10]. However, it remains to be determined whether this increasing trend is also observed in PsA patients or whether pharmacological advances have reduced the need for surgery, as seen in rheumatoid arthritis (RA) [11,12,13,14].

Currently, there is limited and inconsistent literature addressing the rates and outcomes of THA and TKA in PsA patients, particularly in comparison to OA and RA patients.

Additionally, the potential complications, such as infections and delayed wound healing in PsA patients undergoing joint arthroplasty, remain poorly defined, especially given the role of immunosuppressive therapies and chronic inflammation [15,16,17]. As a result, there is a need for an updated review of the current evidence to guide clinical decision-making and optimize patient outcomes. This scoping review aims to fill this gap by synthesizing the available data on the relationship between PsA and orthopedic surgery.

In particular, we aim to address three key questions: (1) What is the risk of undergoing THA or TKA in PsA patients, and how have temporal trends changed over time? (2) What is the prevalence of postoperative complications in PsA patients following THA or TKA? (3) How effective are THA and TKA in improving outcomes for PsA patients, and how do these outcomes compare to those of patients with other forms of arthritis?

By answering these questions, this review seeks to provide a clearer understanding of the risks and benefits of joint arthroplasty in PsA patients.

## 2. Materials and Methods

We conducted this scoping review following the methodological guidelines proposed by the Joanna Briggs Institute [18]. The Preferred Reporting Items for Systematic Reviews and Meta-Analysis extension for Scoping Reviews (PRISMA-ScR) checklist was used to guide the reporting of the review [19]. The protocol was registered through Open Science Framework (OSF) Registries (https://doi.org/10.17605/OSF.IO/45NPT). Consistent with the standards of scoping reviews, no critical appraisal or assessment of risk of bias in the individual studies was performed [18].

### 2.1. Eligibility Criteria and Study Selection

The population, concept, and context (PCC) framework was used to formulate the search question. All studies meeting the following criteria were included in the final review:

- Population: adult patients with a diagnosis of PsA;
- Concepts:
  ○ Risk of undergoing THA or TKA, including changes in the prevalence of PsA patients undergoing THA or TKA over different time periods;
  ○ Safety of THA or TKA in PsA patients, including early and late complications such as bleeding, thromboembolism, surgical site infection, delayed wound healing, loosening, revision, and death;
  ○ Effectiveness of THA and TKA in PsA patients, including reduction in pain and improvement in functional status and quality of life.

- Context: patients above 18 years with a diagnosis of PsA undergoing THA or TKA. Our review sought to include randomized controlled trials (RCTs), quasi-RCTs, prospective or retrospective cohort studies, case–control studies, cross-sectional studies, and qualitative reports.

Studies were selected if they were published in English in international peer-reviewed journals and involved at least 100 PsA patients, except where patient-reported outcomes were the main measure of effectiveness, in which case a minimum of 50 patients was required. We excluded studies if the proportion of patients undergoing TJA was below 50%, or if PsA patients could not be isolated from those with other forms of inflammatory arthritis or OA.

The sample size threshold of 100 patients, or 50 for patient-reported outcomes, was set to ensure sufficient statistical power to draw reliable conclusions given the relatively low prevalence of PsA in comparison to other conditions such as OA or RA. The aim was also to avoid the inclusion of smaller, potentially underpowered studies, which might introduce bias.

### 2.2. Search Strategy

We conducted a comprehensive search in MedLine (via PubMed) and Web of Science (WOS) databases up to 3 February 2024. The search string in MedLine was (“Arthroplasty, Replacement”[MeSH] OR “Arthroplast*” OR “Prosthesis Implantation*”[tw] OR “Joint Prosthesis”[tw] OR “Joint replacement”[tw] OR (“total”[tw] AND (“arthroplast*”[tw] OR “replacement*”[tw]))) AND (“Arthritis, Psoriatic”[MeSH] OR “Arthritic Psoriasis”[tw] OR “Psoriatic Arthritis”[tw] OR “Psoriasis Arthropathica”[tw] OR “Psoriatic Arthropath*”[tw]).

In the WOS, the search string was (Arthroplasty, Replacement* OR Arthroplast* OR Prosthesis Implantation* OR Joint Prosthesis OR Joint replacement* OR (total AND (arthroplast* OR replacement*))) AND (Arthritis, Psoriatic OR Arthritic Psoriasis OR Psoriatic Arthritis OR Psoriasis Arthropathica OR Psoriatic Arthropath*).

Relevant keywords were used for free-hand search, and the bibliographies of selected articles were reviewed for additional sources. Two authors (L.B. and J.C.) designed and implemented the search strategy. A senior investigator (F.U.) supervised the process. No filters were applied regarding the publication date to capture a broad spectrum of studies.

### 2.3. Study Selection and Data Charting

After the removal of duplicate records, two reviewers (J.C. and L.B.) independently screened the titles and abstracts of the retrieved articles. Full-text reading of potentially eligible articles was then performed. Discrepancies between reviewers during the study selection process were resolved through discussion. A senior investigator (F.U.) was consulted if consensus could not be reached.

Each selected article was summarized and the following information was recorded: first author, year of publication, country, study design, time period considered, total number of PsA patients, number of THA or TKA in PsA patients, duration of follow-up, identification of PsA cases, comparator groups, and primary study aim.

### 2.4. Synthesis of Results

We conducted a narrative synthesis of the extracted data, structured around the three objectives of the review:

(1) Risk of undergoing THA or TKA in PsA patients and changes in temporal trends.

In this section, we summarize findings from studies that assess the following:

(1a) The likelihood of PsA patients undergoing THA or TKA compared to the general population;

(1b) The impact of different pharmacological treatments on the risk of THA or TKA in PsA patients;

(1c) The influence of diagnosis timing and inflammatory markers on the likelihood of PsA patients undergoing THA or TKA.

(2) Prevalence of postoperative complications in PsA patients following THA or TKA.

In this section, we summarize the results of studies evaluating the prevalence of medical and surgical complications in PsA patients after THA or TKA compared to those with other conditions such as OA or RA.

(3) Effectiveness and overall outcomes of THA or TKA in PsA patients.

In this section, we summarize the results of studies assessing postoperative pain, function, health status, quality of life, and overall satisfaction in PsA patients following THA or TKA compared to other patients.

## 3. Results

### 3.1. Search Results and Characteristics of the Included Studies

**Figure 1:**
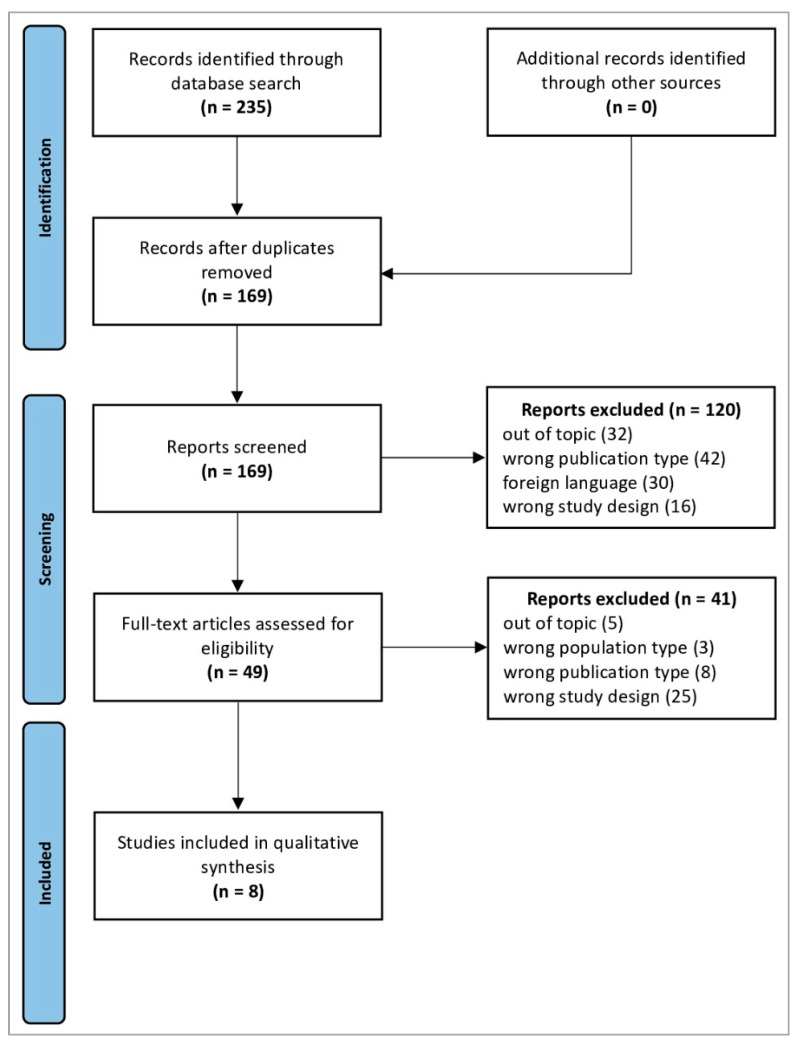
PRISMA 2020 flow diagram. Adapted From [26]. For more information, visit http://www.prisma-statement.org/ (URL accessed on 12 June 2024).

**Figure 2:**
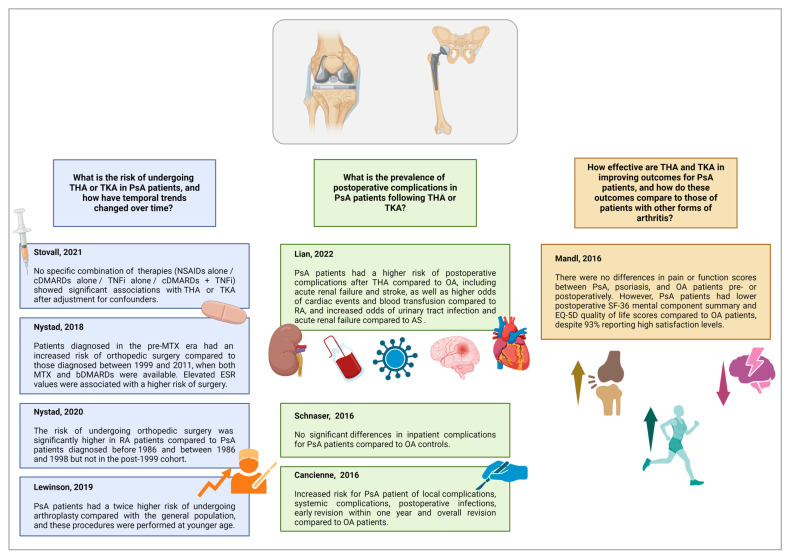
Main results of the studies included in the scoping review. Legend: AS: ankylosing spondylitis; bDMARDs: biological disease-modifying anti-rheumatic drugs; bDMARDs: biological disease-modifying anti-rheumatic drugs; ESR: erythrocyte sedimentation rate; MTX: methotrexate; NSAIDs: non-steroidal anti-inflammatory drugs; OA: osteoarthritis; PsA: psoriatic arthritis; RA: rheumatoid arthritis; THA: total hip arthroplasty, TKA: total knee arthroplasty; TNFis: TNF inhibitors. Created with BioRender.com.

The database search identified 235 studies. After removing duplicates, 169 records proceeded to review; of these, 120 were excluded after screening the titles and abstracts. Full-text evaluation was performed for 49 articles and 8 were included in the qualitative synthesis (Table 1). All included studies were published between 2016 and 2022, with contributions from the United States (*n* = 4), Norway (*n* = 2), the United Kingdom (*n* = 1), and China (*n* = 1). The flowchart detailing the study selection process is shown in Figure 1, while the main results are shown in Figure 2.

**Table 1:** Characteristics of the included studies.

First Author, Year	Country	Study Type	Time Period Considered	Total Number of PsA Patients	Number of THA or TKA in PsA Patients	Duration of Follow-Up	Identification of PsA Cases	Comparator Groups	Primary Study Aim
Cancienne JM, 2016 [20]	United States	retrospective cohort study	from 2005 to 2012	7918	7918 TKA	3 months	ICD codes	153,531 patients with RA; 4575 patients with AS; 1,751,938 patients with OA	to compare the outcomes and complications of TKA performed in patients with inflammatory arthritis with those of TKA performed for OA.
Lewinson RT, 2019 [6]	United Kingdom	retrospective cohort study	from 1995 to 2010	5619	77 THA, 99 TKA	15 years	read codes	5,090,814 patients from the general population	to compare the rates of total arthroplasty between PsA patients and the general population
Lian Q, 2022 [21]	China	retrospective cohort study	from 2005 to 2014	962	962 THA	not reported	ICD codes	17,200 patients with RA; 941 patients with AS; 509,426 patients with OA	to investigate whether perioperative complications in patients with inflammatory arthritis were significantly different from those in patients with OA, and whether patients with different types of inflammatory arthritis also had different perioperative complications.
Mandl LA, 2016 [22]	United States	retrospective cohort study	from 2007 to 2010	63	63 THA	5 years	ICD codes	153 patients with psoriasis; 915 patients with OA	to evaluate short-term outcomes of THA in patients with PsA and those with skin psoriasis, as compared to patients with OA.
Nystad TW, 2018 [23]	Norway	retrospective cohort study	from 1954 to 2011	590	47 THA, 41 TKA	14 years	ICD codes	3 groups of PsA patients stratified into different time periods based on the availability of medical treatments at diagnosis (1954–1985 vs. 1986–1998 vs. 1999–2011)	to investigate the occurrence of orthopedic surgery in PsA patients and whether patient characteristics, treatment, and year of diagnosis affect the need for surgical intervention.
Nystad TW, 2020 [14]	Norway	retrospective cohort study	from 1954 to 2011	590	47 THA, 41 TKA	14 years	ICD codes	1010 patients with RA diagnosed in the period 1972–2009.	to compare RA and PsA patients based on medical treatment received and the frequency of orthopedic surgery, as well as time trends relating to this outcome.
Schnaser EA, 2016 [24]	United States	retrospective cohort study	from 2002 to 2011	3818	3818 THA	not reported	ICD codes	63,550 patients with RA; 4252 patients with AS; 2,018,567 patients with OA; 2496 patients with JIA; 12,555 patients with SLE	to compare perioperative inpatient complications and to determine the most common inpatient complications between the different subtypes of inflammatory arthritis compared with OA patients undergoing primary THA.
Stovall R, 2021 [25]	United States	nested case–control study	from 1994 to 2018	34,512	1003 THA/TKA	1 year	ICD codes	3793 matched controls with PsA	to evaluate whether specific medical treatments were associated with the risk of THA or TKA in PsA.

Legend**:** AS: ankylosing spondylitis; ICD: international classification of diseases; JIA: juvenile idiopathic arthritis; OA: osteoarthritis; PsA: psoriatic arthritis; RA: rheumatoid arthritis; SLE: systemic lupus erythematosus; THA: total hip arthroplasty; TKA: total knee arthroplasty.

### 3.2. Risk of Undergoing THA or TKA in PsA Patients and Changes in Temporal Trends

Four studies addressed the risk of PsA patients undergoing THA or TKA and changes in temporal trends. The findings from these studies are summarized as follows:

**Study by Stovall et al.** [25]**:**

- **Population**: A total of 1003 PsA patients who underwent THA or TKA between 1994 and 2018 compared to 3793 PsA controls who did not undergo surgery. Patients were divided into two groups: those who used non-steroidal anti-inflammatory drugs (NSAIDs) and those who did not. Each group was further divided into four subgroups: (1) NSAIDs or no medication; (2) conventional synthetic DMARD use alone; (3) tumor necrosis factor inhibitor (TNFi) use alone; and (4) DMARD and TNFi use in combination.
- **Key finding**: Patients on NSAIDs or DMARDs alone had lower unadjusted odds of THA or TKA compared to those on TNFis or combination therapy (OR = 0.77, 95% CI 0.61–0.98). After adjustment for confounders, this was no longer statistically significant (adjusted OR = 0.78, 95% CI 0.61–1.00).
- **Statistical significance**: None of the combinations of medical therapies showed significant associations with THA or TKA after adjustments.

**First study by Nystad et al.** [23]**:**

- **Population**: A total of 590 PsA patients were treated between 1954 and 2011, with 117 (20%) undergoing surgery. Of the 171 procedures performed, 53% were arthroplasties (47 THA, 41 TKA). Patients were stratified into three different subgroups: (1) patients diagnosed in the pre-methotrexate era (1954–1985); (2) patients diagnosed when methotrexate was available (1986–1998); and (3) patients diagnosed after both methotrexate and bDMARDs were available (1999–2011).
- **Key finding**: Patients diagnosed between 1954 and 1985 had an increased risk of surgery compared to those diagnosed between 1999 and 2011 (RR = 2.1, 95% CI 1.0–4.2). Elevated ESR values were associated with a higher risk of surgery (RR = 3.4 for ESR 30–59 mm/h, 95% CI 1.8–6.4; RR = 2.4 for ESR ≥ 60 mm/h, 95% CI 1.1–5.0).
- **Statistical significance**: Significant risk associations were found for both diagnosis period and elevated ESR.

**Second study by Nystad et al.** [14]**:**

- **Population**: A total of 590 PsA patients were compared with 1010 RA patients, of whom 31% underwent surgery (41% were arthroplasties). Patients were stratified into three different subgroups: (1) patients diagnosed in the pre-methotrexate era (1954–1985); (2) patients diagnosed when methotrexate was available (1986–1998); and (3) patients diagnosed after both methotrexate and bDMARDs were available (1999–2011).
- **Key finding**: There was a higher risk of surgery in RA patients compared to PsA patients diagnosed before 1986 (*p* < 0.001) and between 1986 and 1998 (*p* < 0.001). No significant difference was observed for those diagnosed from 1999 onwards (*p* = 0.19).
- **Statistical significance**: Significant differences in surgery risk between RA and PsA patients were found in the earlier diagnosis periods, but not in the post-1999 cohort.

**Study by Lewinson et al.** [6]**:**

- **Population**: A total of 5619 PsA patients were compared to 5,090,814 patients from the general population. Among these, 187 first-instance arthroplasties were documented in PsA patients (77 THA; 99 TKA; 11 other joints) and 80,163 in the general population (40,759 THA; 34,410 TKA; 4904 other joints; 90 multiple arthroplasties).
- **Key finding**: PsA patients had an incidence rate ratio (IRR) for arthroplasty of 2.01 (95% CI 1.73–2.34, *p* < 0.0001) and underwent surgery at a younger age (median age 64.7 vs. 70.2 years, *p* < 0.0001).
- **Statistical significance**: Strong statistical significance for increased likelihood and earlier age of surgery in PsA patients.

### 3.3. Prevalence of Postoperative Complications in PsA Patients Following THA or TKA

Three studies addressed the prevalence of postoperative complications in PsA patients following THA or TKA. The findings from these studies are summarized as follows:

**Study by Lian et al.** [21]**:**

- **Population**: A total of 528,529 patients who underwent THA from 2005 to 2014, including 962 PsA patients and 509,426 control patients with OA.
- **Key Findings**: PsA patients had a higher risk of acute renal failure (OR = 1.75, 95% CI 1.16–2.51) and stroke (OR = 2.45, 95% CI 1.40–3.92) compared to OA patients. Compared to RA, PsA patients had lower odds of stroke (OR = 0.46, 95% CI 0.28–0.83) but higher odds of cardiac events (OR = 1.72, 95% CI 1.13–2.79) and blood transfusion (OR = 1.43, 95% CI 1.22–1.68). Compared to AS, PsA was associated with higher odds of urinary tract infection (OR = 2.07, 95% CI 1.05–4.28) and acute renal failure (OR = 2.06, 95% CI 1.08–4.15).
- **Statistical Significance**: Statistically significant increased risks of renal failure, stroke, cardiac events, blood transfusions, and infections in PsA patients.

**Study by Schnaser et al.** [24]**:**

- **Population**: A total of 2,105,238 patients who underwent primary THA from 2002 to 2011, including 63,550 RA patients, 3818 PsA patients, and 2,018,567 OA controls.
- **Key Findings**: PsA was not associated with an increased risk of overall inpatient complications (OR = 1.1, 95% CI 0.9–1.1). PsA patients had a lower risk of gastrointestinal events compared to OA patients (OR = 0.2, 95% CI 0.1–0.6), but no other complications showed significant differences. In contrast, other inflammatory arthritis groups experienced significantly more complications than OA patients.
- **Statistical Significance**: No significant differences in overall inpatient complications in PsA patients compared to OA controls.

**Study by Cancienne et al.** [20]**:**

- **Population**: A total of 1,917,962 patients who underwent primary TKA from 2005 to 2012, including 7918 PsA patients, 153,531 RA patients, 4575 AS patients, and 1,751,938 OA controls.
- **Key Findings**: PsA patients had an increased risk of local complications (OR = 1.3, 95% CI 1.1–1.5), systemic complications (OR = 1.4, 95% CI 1.3–1.5), and postoperative infections (OR = 1.7, 95% CI 1.4–2.0) compared to OA patients. PsA patients also had a higher risk of early revision within one year (OR = 1.7, 95% CI 1.4–2.0) and overall revision (OR = 1.6, 95% CI 1.5–1.8) compared to OA patients.
- **Statistical Significance**: Statistically significant increased risks of local and systemic complications, postoperative infections, and revision surgeries in PsA patients.

### 3.4. Effectiveness and Overall Outcomes of THA or TKA in PsA Patients

One study addressed the effectiveness and overall outcomes of THA or TKA in PsA patients. The findings from this study are summarized as follows:

**Study by Mandl et al.** [22]**:**

- **Population**: A total of 63 PsA patients, 153 patients with skin psoriasis, and 915 OA controls who underwent THA between 2007 and 2010.
- **Key Findings**: There were no differences in pain or function scores between PsA, psoriasis, and OA patients either preoperatively or postoperatively. PsA patients had lower postoperative Short Form-36 (SF-36) mental component summary (MCS) scores compared to OA patients, but physical component summary (PCS) scores were similar across groups. PsA patients had lower postoperative EQ-5D quality of life scores compared to OA patients, but 93% reported high satisfaction levels.
- **Statistical Significance**: Statistically significant lower postoperative MCS and EQ-5D scores in PsA patients compared to OA patients, but no significant differences in improvement in pain or function outcomes.

## 4. Discussion

Our scoping review identified key findings about the relationship between PsA and TKA or THA. While electronic medical records and prospective registries have been used to investigate various aspects, extensive, high-quality evidence remains scarce. This was evident in our review, as we were able to include only eight articles.

We divided the findings into three main research objectives. First, regarding the risk of undergoing THA or TKA and the changes in temporal trends over the years, we found that when considering an extended time frame from 1954 to 2011, a substantially high proportion of PsA patients (20%) needed to undergo orthopedic surgery, with the majority of these interventions being THA or TKA [23]. This is consistent with other registry-based studies reporting high estimates of PsA patients requiring surgery, with rates ranging from 12% to 48% depending on the time period and type of surgery [7,27,28].

Lewinson et al. confirmed that PsA patients had a significantly higher risk of undergoing THA or TKA than the general population, with an incident rate twice as high [6]. Interestingly, PsA patients undergoing these procedures were significantly younger than the general population, further delineating an overall higher risk of joint replacement associated with the disease.

However, the introduction of DMARDs appears to have had a significant impact in reducing the need for THA and TKA [23]. Nystad et al. showed that patients diagnosed in the pre-methotrexate era had a higher risk of undergoing THA or TKA compared to those diagnosed between 1998 and 2011, when both methotrexate and bDMARDs were available [23]. However, Stovall et al. did not find an association between ongoing treatment with DMARDs, including combination therapy with methotrexate and TNFis, and the risk of undergoing THA or TKA [25].

Although the use of TNFis has been shown to inhibit radiographic progression in both RA and PsA, the protective effect of these treatments on reducing the need for joint replacement in PsA remains unclear [29,30,31,32].

Hawley et al. found that the introduction of TNFis significantly reduced the incidence of TKA in early RA patients within the first 5 years after diagnosis [33]. This reduction in TKA incidence was further confirmed by Cordtz et al. and Zhou et al. in their population-based studies [13,34]. Regarding THA, a systematic review and meta-analysis spanning four decades revealed that the global incidence of THA in RA patients decreased from 46/1000 patient-years in 1990–1999 to 11/1000 patient-years in 2000–2009, and further to 7/1000 patient-years in 2010–2019 [35]. The evidence from RA, where studies have demonstrated a significant reduction in THA and TKA rates following the introduction of TNFis, supports the hypothesis that bDMARDs may help prevent joint damage, but this requires further study in PsA.

Elevated inflammatory markers, particularly ESR, were identified as risk factors for THA and TKA. Nystad et al. reported that higher ESR values were strongly associated with an increased risk of surgery, suggesting that patients with higher disease activity may experience progression of joint damage [23]. This was consistent with other studies, such as Kwok et al., who reported that elevated ESR was associated with a 2.4 times higher risk of PsA patients requiring orthopedic surgery [28].

These findings highlight that early and aggressive treatment aiming to control inflammation can potentially reduce the need for joint replacement.

The second objective of our scoping review focused on the prevalence of postoperative complications in PsA patients following THA and TKA. The three included articles presented contrasting results.

Lian et al. found that PsA patients had a higher occurrence of acute renal failure and stroke following THA compared to patients with OA. Additionally, the authors observed a higher risk of acute cardiac events and blood transfusions compared to patients with RA, as well as an increased risk of urinary tract infections and acute renal failure compared to patients with AS [21]. However, Schnaser et al. did not report any significant differences in medical complications between PsA and OA patients, except for a lower risk of gastrointestinal complications [24]. After TKA, Cancienne et al. identified an increased risk of both local and systemic complications in PsA patients compared to OA patients, including postoperative infections, revision surgeries, and higher re-admission rates [20].

The relationship between the use of TNFis and the risk of postoperative infections has been extensively studied in RA, with evidence suggesting an increased risk of infections, particularly in patients with higher disease activity or those on glucocorticoids [36,37,38,39]. However, data on PsA are more limited [40]. Schnaser et al. did not observe an increased risk of infections in PsA patients undergoing THA, but they were unable to account for the details of perioperative medication management [24].

In a registry-based cohort study, Di Martino et al. assessed the risk of revision surgery after THA or TKA in 121 patients with inflammatory arthritis, including 36 with PsA, who received TNFis during the perioperative period [41]. This group was compared to 870 patients with inflammatory arthritis, including 254 with PsA, who were not receiving bDMARDs or tsDMARDs, as well as to 473 propensity score-matched OA patients. The study found that treatment with TNFis did not affect the risk of revision surgery after an average follow-up of 5 years [41].

To interpret the findings of our review in the context of RA, it is important to note that after THA, RA patients face a higher risk of revision (OR = 1.15, 95% CI 1.02–1.29), periprosthetic infection (OR = 1.44, 95% CI 1.29–1.61), and wound infection (OR = 2.15, 95% CI 1.19–3.90) compared to OA patients [42]. However, there are no significant differences in mortality (OR = 1.20, 95% CI 0.89–1.61) or deep vein thrombosis (OR = 0.52, 95% CI 0.12–2.34) between the groups. After TKA, a lower incidence of deep venous thrombosis (OR = 0.84, 95% CI 0.79–0.90) and pulmonary embolism (OR = 0.84, 95% CI 0.78–0.91) has been reported in RA patients compared to those with OA [43].

Interestingly, one of the studies included in our scoping review assessed mortality in PsA patients following THA or TKA [24]. The authors found no significant differences in postoperative mortality rates between PsA and OA patients. This issue was also explored in a large national cohort study of primary THA and primary TKA in the United States, conducted between 1998 and 2014, where PsA patients were analyzed alongside those with axial spondyloarthritis, grouped under the umbrella term of spondyloarthritis (SpA) [44]. The study examined 4,116,484 primary THA and 8,127,282 primary TKA, of which 1.7% of THA and 1% of TKA were performed in SpA patients. Although separate results for PsA patients were not provided, the odds of postoperative mortality were 22% lower after THA and 60% lower after TKA in SpA patients compared to non-SpA controls. However, SpA patients required higher healthcare utilization and experienced a higher rate of in-hospital complications, including longer hospital stays, blood transfusions, and implant infections [44].

The third part of our review examined the effectiveness and outcomes of THA and TKA in PsA patients. Mandl et al. reported that PsA patients achieved similar improvements in pain and functional status compared to those seen in OA patients after THA [22]. Satisfaction levels were also similar across groups. These findings are consistent with evidence from RA, where THA and TKA have been shown to provide significant improvements in quality of life and functional status [42].

Several limitations of our review must be acknowledged. First, the inclusion of only eight studies limits the generalizability of our findings, and the lack of RCTs further reduces the strength of the conclusions that can be drawn. Second, the included studies had different designs, population sizes, and outcome measures, which complicated the synthesis of the results. Third, the use of registry data may have introduced selection bias due to inconsistent diagnostic or coding criteria across different healthcare systems [45]. Follow-up periods were variable among the included studies, and this may have affected the reported outcomes of interest, such as long-term revision rates and postoperative complications. Another limitation further complicating the interpretation of the results is the difficulty in controlling for confounding variables such as comorbidities, differences in perioperative management strategies, and preoperative medication use. More standardized, prospective research would be needed to reliably assess the risks and outcomes associated with THA and TKA in PsA patients.

Furthermore, this review identified several gaps that should be addressed by future research. First, longitudinal prospective studies are needed to assess the impact of bDMARDs and tsDMARDs on the long-term risks of THA and TKA in PsA patients. These studies should be adjusted for disease severity, comorbidities, and medication adherence to delineate the direct effects of treatment. Another research area that has not been adequately explored is the role of inflammation in the early onset of joint damage in PsA and whether early aggressive treatment could reduce the need for THA and TKA. The perioperative management of PsA patients can be complex. Despite the availability of guidelines about the management of immunosuppressive therapies, choosing the optimal timing of DMARD discontinuation and re-initiation and balancing the risk of surgical complications with the prevention of disease flares is a challenge in clinical practice [46]. Research into the comparative effectiveness of THA and TKA between PsA and RA patients may provide insights into the specific surgical needs of PsA patients, helping to tailor perioperative care more effectively.

Finally, we did not conduct a critical appraisal of the included studies. While this aligns with the standards of scoping reviews, we acknowledge that this absence might be considered as a limitation of our study. The lack of formal quality assessment means that the included studies may vary in methodological rigor, which could impact the reliability of the results and the strength of the conclusions drawn. The incorporation of a critical appraisal in future reviews in this area would provide a clearer understanding of the quality and robustness of the available evidence.

As a final remark of our review, a key consideration is the need to strengthen the collaboration between rheumatologists and orthopedic surgeons. Patients with PsA, and those with RA as well, often require orthopedic procedures [47]. However, the latest recommendations from the European Alliance of Associations for Rheumatology (EULAR), the American College of Rheumatology (ACR), and the Group for Research and Assessment of Psoriasis and Psoriatic Arthritis (GRAPPA) do not address the surgical needs of PsA or RA patients [1,48,49,50]. In contrast, the Assessment of SpondyloArthritis International Society (ASAS)/EULAR recommendations highlight the importance of considering potential surgical indications for patients with severe axSpA [51]. This gap raises questions about the current interdisciplinary evaluation of patients and the extent to which shared decision-making between orthopedic surgeons and rheumatologists is integrated into daily practice. Such collaboration is essential for tailoring both operative and non-operative treatment options to improve patient outcomes [52].

## 5. Conclusions

In conclusion, this review highlights that PsA patients have higher rates of THA and TKA, suggesting a potential unmet need in current medical treatment strategies. However, the analysis of temporal trends shows a decreased risk of THA and TKA for patients diagnosed in more recent years, likely due to the growing use of effective DMARDs. Moreover, PsA patients remain at increased risk of postoperative complications, particularly following TKA, including infections and the need for revision surgery. Therefore, the optimization of perioperative care and the close monitoring for postoperative complications are crucial. Despite these risks, PsA patients undergoing THA and TKA can still achieve significant improvements in function and pain relief, which should be communicated to patients considering surgery.

The findings of our scoping review highlight that rheumatologists should focus on early intervention and aggressive management of PsA to prevent joint damage, while perioperative strategies should be aimed at minimizing risks. Researchers should prioritize studies exploring the long-term impact of DMARDs on joint replacement rates in PsA, as well as the development of tailored perioperative protocols to reduce complications.

## Data Availability

All relevant data are presented in the article.
